# The Emulsion of Lymph Orchitic Compound for Stomach Absorption

**Published:** 1910-07

**Authors:** 


### The Emulsion of Lymph Orchitic Compound for Stomach Absorption.

This modification of the original Lymph Compound, for stomach absorption, continues to prove less promptly remedial than its hypodermic equivalent in most diseases, although in a few chronic diseases it has shown equally prompt and equally complete results, as will be seen by reports in this issue.

Case reports indicate that in a few chronic *functional* diseases a more prolonged use of this emulsion may result in nearly as complete reactions. This is particularly true in neurasthenia, melancholia, sexual incompetency, functional and not unusually long standing epilepsy, neurotic asthma, malnutrition, and drug habits.

A few chronic *organic* diseases have apparently responded most decidedly in a large number of cases, but I shall make no positive claims for its value in such diseases until my data are more extensive and conclusive. It seems probable that in the next *Bulletin-Journal* I shall be able to cite positive conclusions regarding its value in chronic rheumatism, infantile paralysis, early hemiplegia, early tabes, neuritis, and possibly a very few other types of organic diseases. It has already done some brilliant work in the diseases named.

One fact seems clear, namely, that exceptin gthe hypodermic preparation, *it is by far the most effective general tonic now in use.*

The best practical proof I can offer of the value of this new preparation follows:

1. It was presented only seventeen months ago.
2. It has never been advertised except in two issues of this *Bulletin-Journal.*
3. Not only in these Bulletins but also in my correspondence, it has been discussed as a largely experimental departure.

4. *In spite of these facts, the "repeat orders" have been over 70 per cent.* The answer is obvious.—*Bulletin-Journal Animal Therapy,* June.

